# Pelvic recurrence after definitive surgery for locally advanced rectal cancer: a retrospective investigation of implications for precision radiotherapy field design

**DOI:** 10.18632/oncotarget.21616

**Published:** 2017-10-07

**Authors:** Chao Li, Yinju Zhu, Tong Tong, Ye Xu, Yun Guan, Jingwen Wang, Huankun Wang, Ji Zhu

**Affiliations:** ^1^ Department of Radiation Oncology, Fudan University Shanghai Cancer Center, Shanghai, China; ^2^ Department of Oncology, Shanghai Medical College, Fudan University, Shanghai, China; ^3^ Department of Radiation Oncology, Dalian Third People's Hospital Affiliated to Dalian Medical University (Dalian Cancer Hospital), Dalian, China; ^4^ Department of Radiology, Fudan University Shanghai Cancer Center, Shanghai, China; ^5^ Department of Colorectal Surgery, Fudan University Shanghai Cancer Center, Shanghai, China

**Keywords:** pelvic recurrence, advanced rectal cancer, radiotherapy, total mesorectal excision, radiation toxicity

## Abstract

**Background:**

To analyze the local distribution of pelvic recurrence after total mesorectal excision, with a view to simplifying the formulation of optimal individualized radiotherapy plans.

**Methods:**

We retrospectively investigated the data of 168 patients diagnosed with recurrent pelvic cancer treated at Fudan University Shanghai Cancer Center between January 2008 and December 2012. The following were collected depending on availability: operative report, histological report, specimen photographs, initial preoperative images, images confirming local recurrence, and clinical history.

**Results:**

A total of 203 lesions of local recurrence were identified. The most common sites of pelvic recurrence were the mesorectum, including the anastomotic stoma in 53.0% of cases; presacral space in 27.4%, and pelvic floor and perineum in 21.4% the cases. Recurrence was most common in the lower pelvic region (i.e., below the upper border of the acetabulum), accounting for approximately 76.2% (128 cases) of cases. In patients with mid-rectal and distal rectal carcinoma,

**Conclusions:**

Patients with pelvic cancer may benefit by individualized treatment plans aimed at achieving a balance between tumor control and minimal risk of irradiation-induced toxicity.

## INTRODUCTION

Multi-disciplinary treatment (MDT) is currently considered the gold standard in the management of locally advanced rectal cancer. Radiotherapy (RT), as a part of MDT, plays an important role in reducing locoregional failure rate and improving, at least to some extent, the survival of patients [1–3]. The benefits of RT have been documented both with [4, 5] and without [6] the use of concurrent chemotherapy. However, these benefits are significantly countered by radiation-induced toxicities of adjacent normal tissue leading to other complications, such as increased risk of perineal wound infection, impaired anal function and fecal incontinence, increased risk of late bowel obstruction, and secondary malignancies. In particular, the volume of the small bowel exposed in the treatment field is a major determinant of the extent of the inadvertent injury to the small bowel [7].

Generally, the choice of the RT protocol in cases of rectal cancer is based on the incidence and predominant site of local recurrence and on the local distribution of the lymphatic system. Some guidelines [8, 18] have been put forth on the contouring of the pelvic regions at risk in RC. However, previous versions of these guidelines were published before the widespread acceptance of total mesorectal excision (TME), which can influence the incidence of pelvic recurrence and the failure rate, depending on the surgical technique applied. Furthermore, the recommendations differ in target definition (clinical target volume (CTV) versus subsites), subsite nomenclature, and anatomical boundaries [8, 18, 10–21].

The superior margin of the pelvic irradiation field is usually marked at the point of the bifurcation of the common iliac vessels into the external and internal iliac vessels, with the approximate boney landmark being the sacral promontory [8]. However, this demarcation leads to the inclusion of the small intestine into the irradiated area. In particular, patients undergoing postoperative RT are likely to experience severe radiation enteritis because of an upward shift of the small intestine after TME.

Therefore, RT fields should target only those areas of the pelvis and perineum that are at a high risk of local recurrence. Reducing the size of the RT portals to cover only these at-risk sites should minimize normal tissue toxicity. This study was aimed at analyzing the local distribution of pelvic recurrence after TME.

## RESULTS

### Clinical and pathological characteristics

After a thorough search for all the medical records and imaging study reports from the database maintained at our center, 962 patients were identified as having pelvic failure after definitive TME surgery. Among these patients, 762 and 32 were excluded due to the lack of useful imaging dataor previous radiotherapy history, respectively. Finally, 168 cases with postoperative pelvic recurrence were included in our study.

The clinical and pathological features of the enrolled patients are listed in Table 1. Among the 168 patients, approximately 56% were men, and the median age of the patients was 57 years (range, 30–86 years). The most common pathological stages were initial pT3 (66.1%) stage and positive lymph node (63.1%). The distance from the anal verge was recorded as a classified variable, in terms of percentage of patients with recurrence at each level: local recurrence at a distance of ≤4 cm, 4–8 cm, and >8 cm was noted in 42.3%, 40.5%, and 13.7% of the cases, respectively. Half the patients had previously undergone anterior resection, and about 80% of the patients had undergone initial surgery at other hospitals. Only about half the patients had received adjuvant chemotherapy. The median interval between the initial surgery and detection of pelvic recurrence was 15 months (range, 3–57 months).

**Table 1:** Demographic and clinical features of all patients

		N
Gender	Male	94
	Female	74
Age (y)	Median	57
	Min–Max	30–86
Initial T stage	T1	4
	T2	24
	T3	111
	T4	26
	Unknown	3
Initial N stage	N0	59
	N1	62
	N2	44
	Unknown	3
Distance from anal verge	≤4 cm	71
	4–8 cm	68
	>8 cm	23
	Unknown	6
Differentiation	Low	82
	Middle	74
	High	5
	Unknown	7
Vascular invasion	Yes	30
	No	131
	Unknown	7
Perineural invasion	Yes	20
	No	140
	Unknown	8
Surgical center	FUSCC	35
	Other hospitals	133
Surgery type	APR	77
	AR	85
	Unknown	6
Adjuvant chemotherapy	Yes	80
	No	88
Interval between surgery and relapse (months)	Median	15
	Min–Max	3–57
Total		168

APR, abdominal-perineal resection; AR, anterior resection; FUSCC, Fudan University Shanghai Cancer Center.

### Patterns of local recurrence

A total of 203 recurrent lesions were observed in the 168 patients (Table 2). The most common sites of pelvic recurrence were the mesorectal area (including anastomotic stoma) in 53.0% of the cases, presacral space in 27.4%, and pelvic floor and perineum in 21.4%. In terms of the pelvic level, recurrence was most common in the lower pelvic region (below the upper edge of the acetabulum), accounting for approximately 76.2% (128 cases) of the cases of pelvic recurrence.

**Table 2:** The distribution of pelvic recurrence site and level of recurrence

		N
Level of recurrence	Upper-pelvis	14
	Mid-pelvis	26
	Lower-pelvis	128
Recurrence site	Mesorectum and anastomotic stoma	89
	Presacral space	46
	Pelvic floor and perineum	18
	Internal iliac area	3
	External iliac area	11
	Inguinal area	36

### Correlation between initial tumor location and location of pelvic recurrence

Table 3 shows the correlation between the site of the primary tumor and the site of local recurrence. Overall, failure in mesorectum/anastomotic stoma and presacral area were common, but this trend was not uniform across all sites of recurrence. In the case of primary tumors in the mesorectum/anastomotic stoma area, the rate of failure was significantly higher for proximal tumors than for distal ones. However, the opposite tendency was noted in the case of recurrence in the presacral space and pelvic floor/perineum.

**Table 3:** Correlation between initial tumor location and site of pelvic recurrence

	≤4 cm	4–8 cm	>8 cm
Mesorectum and anastomotic stoma	27 (38.0)	40 (58.8)	20 (87.0)
Presacral space	23 (32.4)	16 (23.5)	3 (13.0)
Pelvic floor and perineum	21 (29.6)	13 (19.1)	1 (4.3)
Internal iliac area	6 (8.5)	9 (13.2)	2 (8.7)
External iliac area	3 (4.2)	0	0
Inguinal area	11 (15.5)	0	0
Total	71 (100)	68 (100)	23 (100)

*P* = 0.000

Furthermore, some unique distribution patterns of local failure were noted for tumors at certain sites. For primary tumors that were proximal and in the mid-pelvic region, no recurrence was noted in the external iliac and inguinal regions; however, in the case of primary distal tumors, the percentages of recurrence in the external iliac and inguinal regions were 4.2% and 15.5%, respectively. In all these 14 cases (recurrence in the external iliac and inguinal regions in 3 and 11 cases, respectively), the distances of the primary tumors from the anal verge were less than 4 cm. All 3 patients with external iliac failure had an initial pathologic stage of T3N+. No obvious trend was observed in the case of recurrence in the external iliac region and initial pathologic TN stage.

A significant correlation was observed between the primary tumor location and the level of pelvic recurrence. Local failure tended to occur in close proximity to the site of the primary tumor (Table 4). For example, recurrence in the upper pelvis was common in the case of proximal primary tumors, but very rare (<5%) in cases where the distance from the anal verge was ≤8 cm.

**Table 4:** Correlation between initial tumor location and level of pelvic recurrence

	≤4 cm	4-8 cm	>8 cm
Lower-pelvis	68 (95.8)	50 (73.5)	5 (21.7)
Mid-pelvis	2 (2.8)	16 (23.5)	7 (30.4)
Upper-pelvis	1 (1.4)	2 (2.9)	11 (47.8)
Total	71 (100)	68 (100)	23 (100)

*P* = 0.000

### Correlation of the interval between surgery and recurrence to level of pelvic recurrence

Table 5 shows the correlation of the interval between the initial surgery and detection of recurrence to the level of pelvic recurrence. For the lower pelvic region, the risk of recurrence remained similar at different timepoints after surgery. Relapse occurred in 35.9%, 34.4%, and 29.7% of the cases at intervals of ≤1 year, 1–2 years, and >2 years, respectively. However, for the mid- and upper-pelvic regions, the risk of recurrence reduced significantly with increase in the interval (mid-pelvic region: 46.2%, 34.6%, 19.2% and upper-pelvic region: 57.1%, 35.7%, 7.1%, at the three abovementioned time points, respectively).

**Table 5:** Correlation of the interval between initial surgery and pelvic recurrence with the level of pelvic recurrence

	Lower-pelvis	Mid-pelvis	Upper-pelvis
≤1 year	46 (35.9)	12 (46.2)	8 (57.1)
1–2 years	44 (34.4)	9 (34.6)	5 (35.7)
>2 years	38 (29.7)	5 (19.2)	1 (7.1)
Total	128 (100)	26 (100)	14 (100)

P=0.304

## DISCUSSION

This was a retrospective study to evaluate the pattern of pelvic failure after TME in locally advanced rectal cancer. With an understanding of the patterns of pelvic recurrence, we aimed to determine whether the CTV could be optimized and, if so, in what category of patients could the optimization be achieved. Our data showed that mesorectum/anastomotic stoma, presacral area, and pelvic floor/perineum were the most common sites for recurrence of locally advanced rectal cancer after definitive surgery, and different patterns of recurrence were associated with different primary tumor locations. Therefore, we believe that the radiation target volume should be determined on a case by case basis to achieve optimal treatment outcomes.

Although the upper border of the CTV is usually marked by the sacral promontory in many radiotherapy centers worldwide, our data demonstrate that the percentage of recurrence in the upper pelvic region was only 8.3% among all cases of pelvic failure. In particular, for cases of mid- and lower rectal carcinoma, the percentage of recurrence was less than 5%. Similar recommendations favoring the downward shift of the upper margin of the CTV also been put forth previously. Syk et al. reported that 29 of 33 local recurrences were located in the lower two-thirds of the pelvis and that lowering the cranial border of the CTV to 3.5 cm below the sacral promontory would still cover all the cases of pelvic failure [10]. A Swedish research group analyzed images of 83 local failures obtained from a population-based cohort of 2,315 patients and noted that all failures were located in the lower 75% of the pelvis, which is anatomically below the S1-S2 intervertebral space [11]. In a subsequent three-dimensional analysis of recurrence pattern, the investigators concluded that the cranial border of RT field could safely be lowered for patients without expected nodal or circumferential resection margin involvement [12]. Another Dutch study [13], which reviewed images from 70 rectal cancers with local failure, also found that most failures were located in the lower pelvis, and only 5 cases of recurrence occurred at the level of S1-S2. Thus, these results are consistent with those of our present study. We believe that it is reasonable to recommend that the upper pelvis, defined from the base of L5 to the base of the sacroiliac joint, may be omitted from CTV in selected patients with distal tumor location, rather than defining an unchangeable standard.

In the light of the abovementioned considerations, a recent update of Roel's guidelines discusses the possibility of modifying the cranial level of the CTV [14]. Before the era of TME, surgery alone was associated with a high local-regional failure rate [15, 16]. However, with the introduction of TME surgery, the local-regional recurrence rate declined significantly. Traditional guidelines may no longer be suitable. However, the recommendation is consistent with the post-TME era data on local recurrences, which indicate that the rate of recurrence is the highest in the posterior pelvis and anastomotic area [17] and that radiological evidence of recurrence in the lateral lymph node (LLN) is well below 5%[11].

Further, in the recent update of the consensus guidelines published for rectal cancer [18], one of the main updates is the level of the LLN cranial border. In the previous guideline, the cranial border was defined as the level of the bifurcation of the common iliac arteries into the external and internal iliac arteries, which in terms of bony markers coincides with the promontory [14]. Now, in case of cT3 and cN0 tumors without invasion of the mesorectal fascia, the upper border of the LLN has been lowered to the cranial border of the mesorectum, which corresponds to the level of the bifurcation of the superior rectal artery; this is consistent with our findings.

With respect to the inguinal and external iliac regions, the consensus [18] was that the external iliac region should be included in the treatment area, if the primary tumor invades adjacent organs (cT4) or if the anterior LLNs (or obturator nodes) are involved [14, 19].

As per the recommendations, inguinal regions are not commonly involved in rectal cancer, except in case of positive inguinal nodes, massive tumor extension into the internal or external anal sphincter, or infiltration of the lower third of the vagina [13, 20–21]. In our study, none of the patients with primary tumors in the mid- and proximal-rectal regions developed recurrence in the external iliac and inguinal regions, despite the fact that some patients had stage pT4 disease. Among patients with initial distal rectal cancer, only 3 and 11 had recurrence in the abovementioned regions (4.3% and 15.5%, respectively, irrespective of the whether the tumor stage was T3 or T4). Therefore, we inferred that the conventional protocol of irradiation of the external iliac and inguinal regions may not be reasonable in cases of primary proximal and mid-rectal tumors. Even in cases of distal rectal adenocarcinoma, chances of over-treatment may be high because of the low recurrence rate.

This study does have a few limitations, including some of the inherent inadequacies of retrospective investigations. First, the study population included cases of pelvic recurrence of rectal cancer, rather than a prospective cohort of patients who received definitive TME surgery. Therefore, in our study, only the characteristics of local recurrence could be assessed, and not the exact rate of pelvic failure. Although the percentage of recurrence at a given site could be partly inferred in terms of the overall recurrence rate, the values were not always consistent. Secondly, about 80% of the patients in our study population received initial definitive surgery at other hospitals, and half did not receive adjuvant chemotherapy, despite locally advanced rectal cancer. Thus, the quality of initial treatment administered to the patients may be questionable in some cases because of the lack of availability of sufficient data from the patient's medical records.

**Figure 1:**
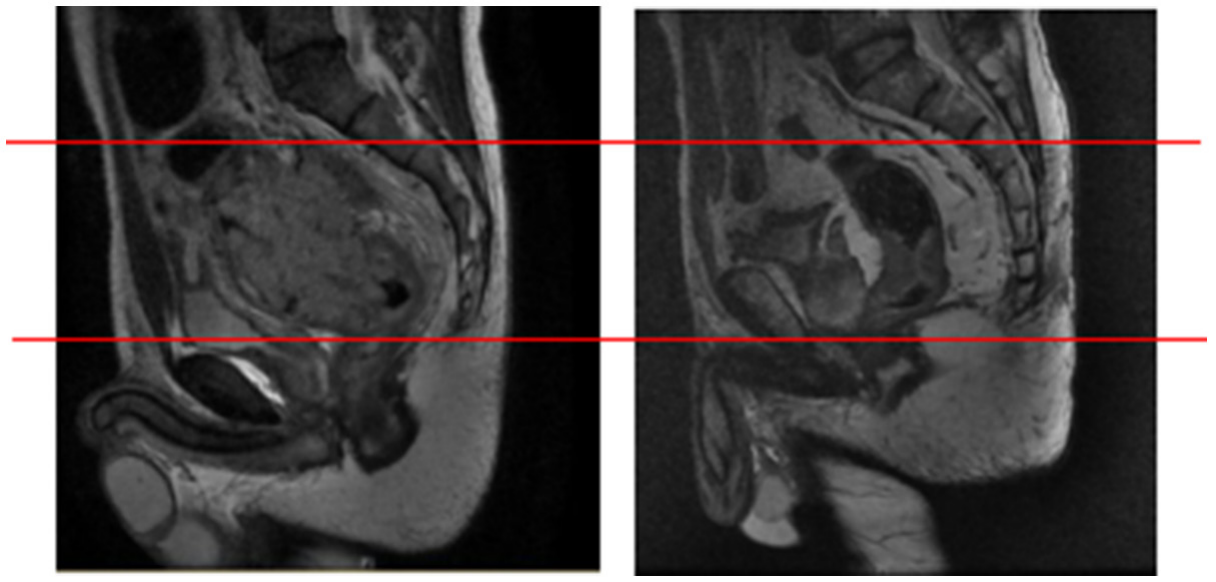
Representative images of two patients with pelvic recurrence Representative images of two patients who had tumors that were almost equidistant from the anal verge. Both patients showed totally different failure patterns, with recurrence occurring at different pelvic sites.

Thirdly, the location and size of the primary tumor are important determinants of the site of pelvic recurrence. For instance, as shown in Figure 1, two patients whose records showed that the tumors were almost equidistant from the anal verge showed totally different failure patterns. Therefore, some modifications are essential when applying our recommendations in clinical practice. In cases of distal rectal tumors, the upper pelvis could be omitted for cases in which the upper border of the gross tumor was more than 2 cm away from the base of the sacroiliac joint. This hypothesis will be validated in the further prospective studies.

## MATERIALS AND METHODS

### Patient selection

This study was a retrospective investigation of data collected for all patients who were diagnosed with pelvic recurrence of rectal cancer at Fudan University Shanghai Cancer Center between January 2008 and December 2012. The criteria for inclusion were as follows: history of primary rectal cancer, with distance from anal verge <12 cm; histologically confirmed adenocarcinoma; history of definitive TME surgery; initial pathological stage T3 or T4 and/or N+; no history of initial distant metastases; no history of previous RT, before or after TME; and availability of images indicating the precise site of recurrence. Patients with a history of any of the following were excluded: ischemic heart disease; inflammatory bowel disease; malabsorption syndrome; peripheral neuropathy; or psychological disorders.

Local recurrence was diagnosed by radiologic evidence of any infiltrative, expansive, or asymmetric pelvic mass with some degree of contrast enhancement that could not be explained by normal or postoperative changes. All patients with local recurrence were reviewed individually. Data were collected from the following patient records, if they were available: operative report, histological report, specimen photographs, initial preoperative imaging, imaging evidence of local recurrences, and clinical history. The data were reviewed on a case-by-case basis by 2 radiation oncologists (J Zhu and YJ Zhu), one radiologist (T Tong), and one surgeon (Y Xu).

### Data management

The collected data comprised demographic and clinical-pathological features (e.g., age, gender, initial T and N stages, distance from the anal verge, type of surgery, and surgical center), as well as characteristics of recurrence (precise anatomical site and interval between initial surgery and pelvic failure). The identified precise locations of pelvic recurrence included the mesorectum and anastomotic stoma, presacral space, pelvic floor and perineum, internal iliac area, external iliac area, and inguinal area. For patients with recurrence at multiple pelvic sites, all sites were recorded individually.

The level of pelvic relapse was classified as follows [9]. The upper pelvis was defined as the region from the base of L5 to the base of the sacroiliac joint. The mid-pelvis was the region between the base of the sacroiliac joint to the upper edge of the acetabulum. The lower pelvis was considered the area below the upper edge of the acetabulum. For patients with recurrence at more than one pelvic site, the level of pelvic recurrence was defined according to the location of the larger lesion.

### Statistical analysis

The patient characteristics were described by frequency and percentage of classified variables, by mean and standard deviations for normally distributed continuous data, and by the median, minimum, and maximum for non-normally distributed continuous data. Pearson's chi-squared test and Fisher's exact test were used to evaluate associations between categorical variables. A P-value of < 0.05 was considered statistically significant.

## CONCLUSION

Most pelvic recurrences occurred in the lower pelvis. Less than 5% of the cases of relapse in the upper pelvis were observed in cases of mid- and distal-rectal carcinoma. Recurrence in the external iliac and inguinal area was rare in patients with distal rectal cancer. These findings suggest that individualized treatment plans need to be drawn for patients to achieve a balance between tumor control and reduced risk of irradiation-induced toxicity and thereby achieve improved treatment outcomes.

## Abbreviations

MDT: Multi-disciplinary treatment
RT: Radiotherapy
TME: Total mesorectal excision
CTV: Clinical target volume
LLN: Lateral lymph node
